# The importance of burrowing, climbing and standing upright for laboratory rats

**DOI:** 10.1098/rsos.160136

**Published:** 2016-06-29

**Authors:** I. Joanna Makowska, Daniel M. Weary

**Affiliations:** Animal Welfare Program, University of British Columbia, 2357 Main Mall, Vancouver, British Columbia, CanadaV6T 1Z4

**Keywords:** housing, environmental enrichment, natural behaviours

## Abstract

Standard laboratory cages prevent rats (*Rattus norvegicus*) from performing many behaviours that they perform in the wild, but little is known about how this may affect their welfare. The aims of this study were (i) to record the propensity to burrow, climb and stand upright in 3-, 8- and 13-month old laboratory rats housed in semi-naturalistic environments and (ii) to compare the frequency of lateral stretching in semi-naturalistic versus standard-housed rats; we predicted standard-housed rats would perform more lateral stretches to compensate for the inability to stretch upright. Rats' propensity to burrow remained constant as they aged (approx. 30 bouts per day totalling 20–30 min), suggesting burrowing is important to rats. Climbing decreased from 76 to 7 bouts per day at 3 versus 13 months, probably because of declining physical ability. Upright standing decreased from 178 to 73 bouts per day, but continued to be frequently expressed even in older rats. Standard-housed rats stretched much more frequently than semi-naturalistic-housed rats (53 versus 6 bouts per day at 13 months), perhaps in compensation for inability to stretch upright and to relieve stiffness caused by low mobility associated with standard housing. These findings suggest that standard laboratory cages interfere with important natural behaviours, which is likely to compromise rat welfare.

## 1. Introduction

The breeding of rats (*Rattus norvegicus*) for the purpose of experimentation began in the 1840s in Europe, making rats the first mammalian species to be domesticated primarily for scientific purposes [1]. The foundations for laboratory rat husbandry were laid down by researchers at The Wistar Institute of Anatomy and Biology in Philadelphia who, beginning in 1906, conducted research into ‘the means of making [rats] contented and happy’ [2] to enable them to develop appropriate housing and ancillary equipment [3]. As a result of their research, the cages designed by The Wistar Institute contained, among other things, a substrate that allowed rats to burrow, and a large, 53 cm diameter running wheel [2,4].

The Wistar Institute's early cage was chiefly designed with rats' welfare in mind, but other models prioritized low costs and ease of cleaning [4,5]. Today's standard laboratory cages offer rats little opportunity to perform many behaviours that are part of their repertoire in the wild, such as burrowing and climbing. Standard cages also prevent rats from standing upright: current regulations in the European Union [6], the United States [7] and Canada [8] mandate a minimum cage height of 18–20 cm, but rats stand at a height of about 22 cm by 2.5 months of age and 26–30 cm by the time they are fully grown [9,10]. In general, housing animals in enclosures that restrict freedom of movement and ability to fully extend limbs is regarded as unacceptable by the public [11–13]. This mounting public opposition has led to regulatory changes in the way many farm animals are housed, including the ban on battery cages for chickens in the European Union, and new legislation in several US states prohibiting the housing of animals without the ability to stand up or extend their limbs without touching the sides of their enclosure [14].

In the wild, rats construct and live inside burrows that they expand and modify frequently [15,16]. Rats are also adept climbers and use this behaviour to escape from predators and to forage [17,18]. Norway rats have been observed to climb up trees, thicket and dry stalks to forage for berries and grain [16,19]. They stand upright as they explore and socialize with other rats [20].

Despite more than 150 years of captive breeding, laboratory rats who are placed in a more naturalistic environment still perform these and other behaviours from their wild ancestors' repertoire [21–23]. Domestication does not seem to have eliminated any behaviours, although in some cases it may have altered the quality and thresholds needed to initiate them [24,25]. For example, when given the opportunity, laboratory rats readily burrow and climb, but burrows tend to be less complex [26,27] and climbing bouts are shorter and less frequent [18] in domesticated laboratory rats versus wild Norway rats.

Although it is known that laboratory rats readily engage in burrowing, climbing and upright standing, there is little information regarding how important these behaviours are to rats. As a starting point, some information could be gained by investigating rats' propensity to perform these behaviours and how these change over the course of the animal's development. Rats, like many animals including humans, spend less time in ambulatory activity and exploration and more time resting as they age [28–32]. Arguably, more weakly motivated activities will be traded for rest as animals age, while strongly motivated activities will continue to be performed. However, a special case should be made for activities that require a high degree of physical aptitude: ageing is associated with loss of muscle strength, coordination and balance, so the performance of more physically challenging activities may decline because of physical inability rather than low motivation.

Animals must decide how much time to allocate to different behaviours, and if the total daily active time decreases, then the cost of performing any of the individual behaviours may increase [33]. According to this perspective, behaviours that are important to an individual will continue to be performed even as the total time available declines; such behaviours can be considered to have ‘inelastic demand’ [33–35].

To our knowledge, no study has investigated the frequency, duration or distribution across time of burrowing behaviour in wild or domesticated rats. With respect to climbing, one study reported that male laboratory rats aged 7–8 months climbed an average of 0.2 times and for 0.7 s, and females climbed 1.1 times and for 27.4 s, when placed into an unfamiliar enclosure for 15 min during the light phase of the light–dark cycle [18]. The propensity to climb probably differs in a novel versus home environment, and in the light versus the dark phase, so drawing conclusions about the importance of this behaviour in rats' daily life based on these results is difficult. Finally, two studies investigated upright standing in the rat. The first recorded the amount of time 6-month old rats spent in upright standing over the course of 5 days and found that rats spent on average 5–14% of daily active time standing taller than 22 cm, and 3–6% time standing taller than 27 cm [9]. No information was given on the frequency or temporal distribution of upright standing. The second tested the proportion of time large males spent in cages that were 16.8 cm versus 23 cm high, and found no preference for one cage over the other [36]. However, even a height of 23 cm would not allow a large rat to stand fully upright, so this study tested rats' preference for increased vertical space rather than the ability to stand upright. If individual bouts of upright standing were brief, then even if rats used this cage frequently to stand (but for short periods of time), this may not have necessarily translated into more frequent use. Indeed, in a barren cage, rats may prefer the lower cage for resting and the taller cage for exploring and stretching.

The goal of this study was to investigate whether laboratory rats are motivated to perform certain natural behaviours that are not possible in standard cages; if such evidence is found here, then further research should address the specific welfare benefits of allowing, or consequences of preventing, rats from engaging in each of these behaviours. Thus, the first aim of this study was to describe the daily frequency, total duration and distribution throughout the day of burrowing (excavation of burrows), climbing and upright standing in laboratory rats reared in semi-naturalistic cages. This was done at three different ages (3, 8 and 13 months old) to capture developmental changes as rats age. Previous work has shown that at 3 months of age rats are at their most active [37–39] and that by 8 months of age they have become socially mature, a stage associated with changes in behaviour [40].

Stretching—formally referred to as pandiculation—is an innate behaviour that occurs in similar form and context across a wide range of species [41,42]. Rats stretch in the vertical position while standing upright and also in the lateral position while remaining parallel to the ground. Because standard-housed rats are unable to stretch in the upright position, we hypothesized that they would perform more lateral stretches than their semi-naturalistic-housed counterparts to compensate for the inability to stretch vertically. Therefore, the second aim of this study was to record the frequency of lateral stretching in 13-month old laboratory rats reared in standard versus semi-naturalistic cages.

## 2. Material and methods

### 2.1. Animals and housing

Forty-two, 22- to 23-day-old female Sprague–Dawley rats were purchased from Charles River Laboratories Canada. As soon as they arrived at our facility, they were systematically assigned to either semi-naturalistic cages (six cages each housing five rats) or standard cages (six cages each housing two rats). In assigning rats to housing treatment, the experimenter alternated between semi-naturalistic and standard cages, and within each cage alternated between rats huddled at the back of the shipping box and those who reared at the front.

All cages were in one room. Rats were housed under reversed lighting, with lights off from 11.00 to 23.00 h. Mean (±s.d.) room temperature and humidity were 23.9 ± 0°C and 44.5 ± 10.6% during data collection at 3 months of age; 24.0 ± 0°C and 20 ± 0% at 8 months of age and 21.6 ± 0°C and 66.5 ± 2.1% at 13 months of age.

**Figure 1.**
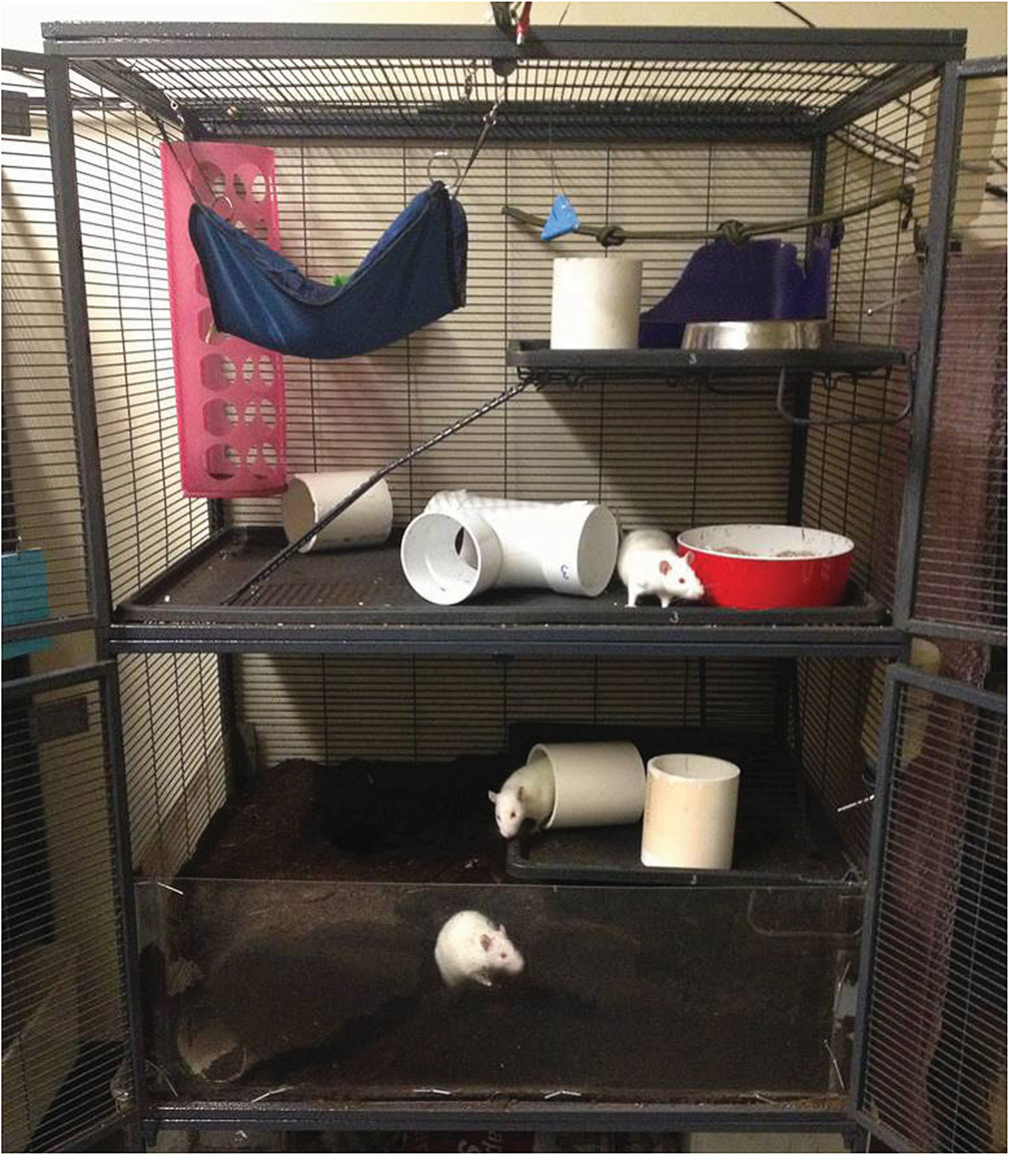
Photograph of a semi-naturalistic cage. Cages were split into four levels connected by ramps. Each cage was furnished with litter boxes, several PVC pipes, a climbing structure, a hammock and a horizontal rope across the top floor. The bottom level was filled with soil substrate.

Semi-naturalistic cages (figure 1; Critter Nation™ double unit with stand, MidWest Homes for Pets, Muncie, IN, USA) were made of horizontal galvanized wire bars to enable climbing, and measured 91 × 64 × 125 cm (L × W × H). The lower portion of each cage was lined with Plexiglas so that the bottom 30 cm of the cage could be filled with a mixture of black earth, compost and sphagnum peat moss (3-in-1 Landscape Soil, Premier LiteWay, Rivière-du-Loup, QC, Canada). This soil substrate was watered every few days to prevent it from drying out and causing burrows to collapse [21]. Burrow construction and maintenance caused soil to fall outside of the cage, so fresh soil was added as needed to maintain levels. Rats had ad libitum access to rat chow (LabDiet® 5012, PMI® Nutrition International, LLC, Brentwood, MO, USA) and tap water, but their diet was supplemented several times per week with unsweetened cereal, nuts or seeds.

Standard cages were made of polycarbonate and measured 45 × 24 × 20 cm (L × W × H). Each cage contained aspen chip bedding (Northeastern Products Corp., Warrensburg, NY, USA), one PVC pipe measuring approximately 18 × 10 cm (L × diameter), and two pieces of brown paper towel. Rats had ad libitum access to rat chow and tap water.

### 2.2. Data collection

Cages were filmed continuously with infrared security monitoring cameras (Swann SWDVK-162608; resolution: 480 TVL) mounted to face each cage. Each age period was defined as lasting two weeks from the day rats turned the target age; for example, the period ‘3 months’ was defined to be when rats were 3 to 3.5 months old. At each age period, we identified a subset of days when there were minimal husbandry procedures (e.g. no cage cleaning) or other disturbances (e.g. no adding soil). From each subset, we randomly selected two days for analysis; half the cages at each age period were scored on each of these two randomly selected days. At 3 months of age, only four cages were scored instead of six because of missing video files. Lateral stretching in standard cages was scored only at 13 months of age.

Within each age period, we randomly selected one semi-naturalistic cage to be scored continuously for the full 24-h period. We then sampled the resulting 24-h data at different intervals and durations to determine a sampling method that predicted the actual frequency and duration of each behaviour with more than 80% accuracy in at least two of the three cages sampled, and used this sampling method to score the remaining cages (see Results). The sampling methods that were tried were a rate of 33% (20 min every 1 h; 5 min every 15 min; 2 h before and 2 h after lights turn on and off); 50% (every other hour; 30 min every 1 h; 15 min every 30 min; 10 min every 20 min; 5 min every 10 min; 3 h every 6 h; 4 h every 8 h), 66% (2 h every 3 h; 40 min every 1 h) and 75% (3 h every 4 h). If more than one sampling method satisfied our criteria, then the method requiring lower sampling rate was chosen; if sampling rate was the same (e.g. 66%) between the methods satisfying our criteria, then the method allowing more continuous watching was chosen (e.g. 2 h every 3 h chosen over 40 min every 1 h). We could not assume that the frequency and temporal distribution of lateral stretching would be the same in the standard cages (indeed, we hypothesized that it would be different) so we also scored three randomly selected standard cages for a full 24-h period to determine a sampling method that would predict the frequency of lateral stretching in standard cages with the same accuracy criteria as used for the semi-naturalistic cages (i.e. more than 80% accuracy in at least two of the three cages), and used this sampling method to score the remaining three standard cages.

We recorded the start and end time of each occurrence of the target behaviours (table 1) [43]. Rats were unmarked and could not be identified as individuals; therefore, frequencies and durations were scored collectively for the whole cage, and this total was divided by the number of rats in the cage to obtain mean values per rat. All semi-naturalistic cages housed five rats at 3 months of age, but by 8 months two rats had been removed from the study for health reasons, so two semi-naturalistic cages housed four rats instead of five. By 13 months of age, one standard-housed rat was removed from the study for health reasons, so one cage housed one rat and the others housed two rats each.

**Table 1.** Behaviours scored and their definitions. All four behaviours were scored in semi-naturalistic cages; lateral stretching was also scored in standard cages (the other three behaviours were not possible in the standard cage).

behaviour	definition
burrowing	rat is displacing soil using fore legs and/or kicking out with the hind legs
climbing	rat is suspended with all paws in contact with a vertical surface or the cage ceiling
upright standing	rat is upright; hind legs are extended and fore paws are either unsupported (rare) or resting on a vertical surface (common); back is either straight or slightly arched (concave)
lateral stretching	rat is parallel to the ground with the body elongated and back slightly arched; head and tail often angled upwards; hind legs and sometimes one fore leg are outstretched; rat is often yawning

Burrowing frequently occurs in bouts, with rats repeatedly digging their way into the burrow (and out of view) and reappearing some moments later pushing soil out with the fore paws; once at the surface rats quickly turn around and repeat the sequence ([16]; personal observations of rats working on tunnels formed along the Plexiglas wall). Therefore, scoring each time the rat was burrowing at the soil surface as a separate event overestimated the frequency and underestimated the duration of burrowing. For a more accurate estimate of the frequency and duration of burrowing, rats were scored as engaged in one burrowing event if they burrowed their way into a burrow *and* were still burrowing when they re-emerged (pushing soil out of the burrow with their fore paws). On rare occasions, rats burrowing their way into a tunnel did not emerge for several minutes. In these rare cases, the burrowing event was considered finished if a rat failed to re-emerge within 4 min. This criterion was based on observations of rats who could be seen burrowing inside tunnels built along the Plexiglas wall at the front of the cage.

Two experienced observers scored behaviours in the semi-naturalistic cages, each scoring half of the cages at each age period. To determine inter-observer reliability for burrowing, both observers scored a set of 12 randomly selected 2 h clips; for climbing, upright standing and lateral stretching in the semi-naturalistic cages, both observers scored a set of 30 randomly selected 5 min clips (see subsection on sampling method in the results section for rationale). Only one observer scored lateral stretching in the standard cages, so reliability was not tested for this behaviour in this housing system.

### 2.3. Statistical analysis

To determine inter-observer reliability, a Pearson correlation coefficient (SAS v. 9.4) was calculated on the 12 (burrowing) or 30 (climbing, upright standing, lateral stretching in the semi-naturalistic cage) pairs of data. After performing visual inspection of residuals to verify normality and homogeneity of variances, the overall effect of age on the frequency and duration of each behaviour was calculated using a mixed model that included age as a repeated measure. Comparisons between individual pairs of age conditions were not performed, because we were not specifically interested in differences between particular ages. The frequency of lateral stretching between semi-naturalistic and standard cages at 13 months of age was compared using an independent samples *t*-test with the Satterthwaite variance estimator method for unequal variances.

## 3. Results

### 3.1. Sampling method

One sampling method satisfied our criteria for burrowing, and this was a rate of 66% and consisted of scoring continuously for 2 h, every 3 h during the 24-h period. For all other behaviours in the semi-naturalistic cages, two methods satisfied our criteria; one was a rate of 33% and consisted of scoring continuously for 5 min, every 15 min, and the other was a rate of 50% and consisted of scoring continuously for 10 min, every 20 min. The former method was selected because it required lower sampling rate. Accuracies (estimated with the sampling method relative to what was measured in the full 24-h period) provided by each sampling method are presented in table 2.

**Table 2.** Accuracy^a^ (%) of the sampling methods used in the semi-naturalistic cages.

	frequency			duration		
	age (months)			age (months)		
behaviour	3	8	13	3	8	13
burrowing	116	110	95	105	113	119
climbing	102	97	104	107	117	117
upright standing	97	100	107	92	111	112
lateral stretching	88	70	88	84	64	88

^a^Values represent the estimated frequency and duration relative to the full 24-h sample; accuracy = estimated value/sampled value × 100.

The only sampling method that satisfied our criteria for lateral stretching in the standard cages was a rate of 75% and consisted of scoring continuously for 3 h, every 4 h during the 24-h period (table 3).

**Table 3.** Accuracy^a^ (%) of the sampling method used in the standard cages.

	frequency			duration		
	cage			cage		
behaviour	1	2	3	1	2	3
lateral stretching	95	108	99	99	109	105

^a^Values represent the estimated frequency and duration relative to the full 24-h sample; accuracy = estimated value/sampled value × 100.

### 3.2. Inter-observer reliability

Pearson correlation coefficients were 0.98 for the frequency and 0.99 for the duration of burrowing; 0.96 for the frequency and 0.90 for the duration of climbing; 0.99 for the frequency and 0.97 for the duration of upright standing; and 0.81 for the frequency of lateral stretching in the semi-naturalistic cages. While both observers recorded a very similar frequency of upright standing in each 5-min sample, when specific occurrences recorded by both observers were compared, each observer missed approximately 15% of the observations from the combined total. This means that while the frequencies provided by each observer did not differ, both observers tended to underestimate the total number of events of standing upright by at least 15%. Similarly, the frequency of climbing was underestimated by 10–15%.

### 3.3. Main study

**Figure 2.**
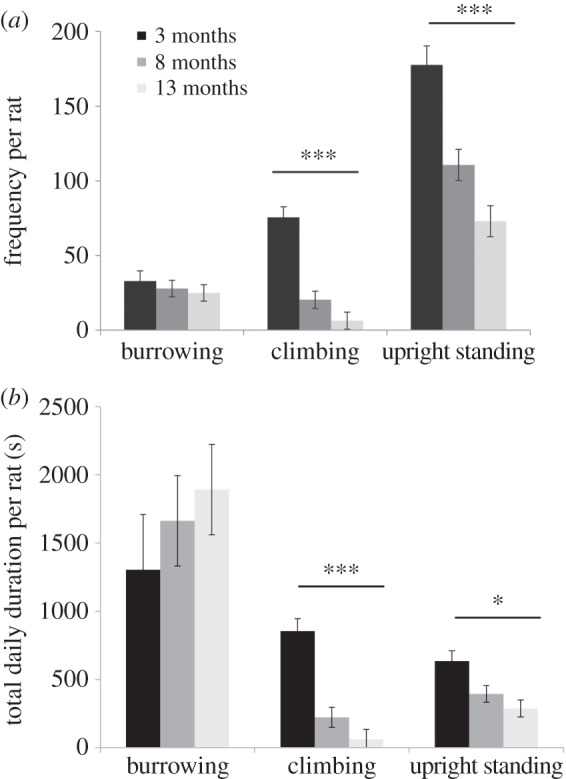
Lateral stretching frequency (*a*) and duration (*b*) of burrowing, climbing and upright standing per day per rat at 3, 8 and 13 months of age in semi-naturalistic cages. Data are mean ± s.e. based on four cages housing five rats at 3 months, and six cages housing five (*n* = 4) or four (*n* = 2) rats at 8 and 13 months; *p < 0.05 and ***p < 0.001.

**Figure 3.**
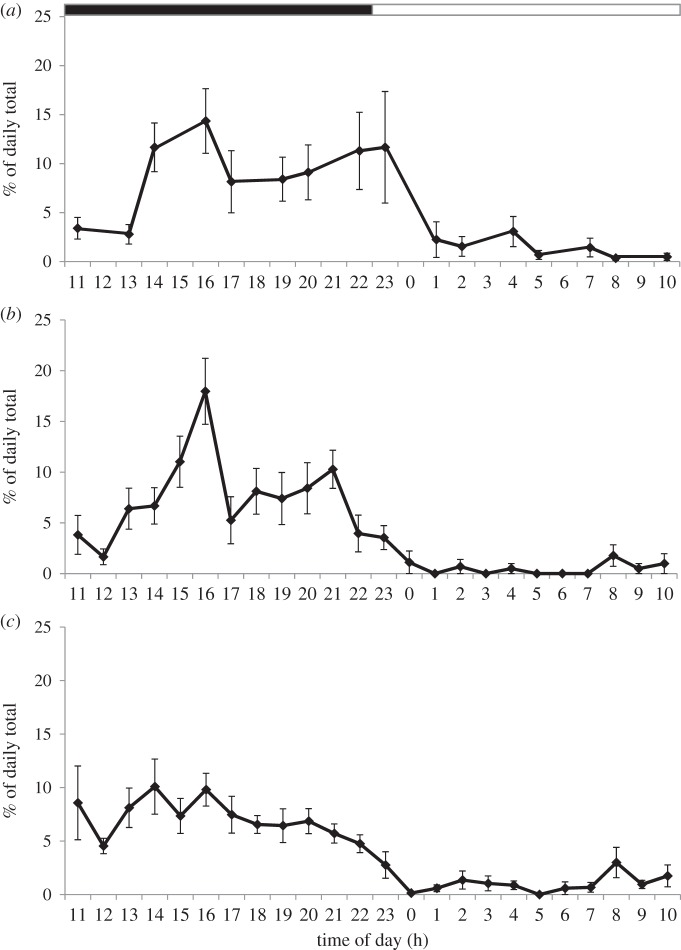
Distribution of burrowing (*a*), climbing (*b*) and upright standing (*c*) throughout the day at 8 months of age. The dark period was from 11 to 23 h. Data represent mean ± s.e. based on values obtained from six cages housing five (*n* = 4) or four (*n* = 2) rats.

Videos showing examples of the behaviours reported here can be seen in the electronic supplementary material. Burrowing, climbing, upright standing and lateral stretching were performed in every cage and at every age (figure 2). The frequency (*F*_2,8_ = 0.41, *p* = 0.6799) and duration (*F*_2,8_ = 0.63, *p* = 0.5563) of burrowing did not vary with age. Climbing and upright standing declined with age in both frequency (climbing: *F*_2,8_ = 30.49, *p* = 0.0002; upright standing: *F*_2,8_ = 20.52, *p* = 0.0007) and duration (climbing: *F*_2,8_ = 24.98, *p* = 0.0004; upright standing: *F*_2,8_ = 6.30, *p* = 0.0228). All behaviours were expressed throughout the 24-h period, but at much higher frequencies during the dark phase (figure 3).

The duration of burrowing bouts varied from approximately 1 s to 13 min; approximately 25% of the bouts lasted longer than 1 min (table 4). Approximately 20–30% of climbing bouts lasted 1 to 2 s, with maximum bout duration of 3 min in 3-month old rats, declining to 40 s in 13-month old rats. The majority of bouts of upright standing were brief, with approximately 70% of bouts lasting just 1–3 s.

**Table 4.** Range and median duration (s) of all bouts of burrowing, climbing and upright standing at each age.

	3 months old		8 months old		13 months old	
behaviour	range	median	range	median	range	median
burrowing	1–435	16	1–693	26	1–967	27
climbing	1–166	5	1–97	5	1–39	6
upright standing	1–127	2	1–137	2	1–32	2

Rats burrowed even though several tunnels were already present, and the conformation of tunnel entrances as seen from the soil surface changed on an almost-daily basis. Frequently, rats who were engaged in other activities in the upper levels of the cage would suddenly run down to the soil to begin burrowing, sometimes bounding in and out of the burrow during and after a burrowing bout.

Many climbing events seemed to occur as a means of moving from one location to another (see electronic supplementary material, video S1). In these instances, rats chose to climb rather than to take a longer route. Other climbing bouts seemed to serve an exploratory function: rats would start out rearing, then would jump up and climb the cage wall as high as it allowed; after some seconds in this suspended position, they would climb down near to where they started (see electronic supplementary material, video S2). We observed a few instances of rats using the cage ceiling as ‘monkey bars’, i.e. swinging from the ceiling by their fore limbs.

Most occurrences of upright standing seemed to serve an exploratory function: rats' heads were angled upwards with indications that they were sniffing (e.g. slight up-down head movements; see electronic supplementary material, video S3). Rats also stood upright to stretch; on these occasions, the back was arched, one fore limb was often outstretched and rats usually threw their head back and yawned (see electronic supplementary material, video S4). When scoring upright standing, we noted if the event appeared to serve the purpose of exploration or stretching. However, the reliability of differentiating these two types of upright standing was poor so we do not report the results. However, ‘exploratory’ bouts of upright standing appeared to comprise about 80–90% of occurrences of upright standing, and upright stretching comprised about 10–20%.

Rats housed in the semi-naturalistic environment performed lateral stretching (means ± s.e.m.) 9.2 ± 3.18 times a day at 3 months; 13.0 ± 2.59 times a day at 8 months; and 6.4 ± 2.59 times a day at 13 months. This difference was not statistically significant (*F*_2,8_ = 1.65, *p* = 0.2513). Duration of lateral stretching also did not vary with age (*F*_2,8_ = 1.82, *p* = 0.2225; 14.5 ± 5.42 s per day at 3 months; 23.5 ± 4.42 s at 8 months and 12.0 ± 4.42 s at 13 months). Thirteen-month old standard-housed rats performed lateral stretching much more frequently (mean ± s.e. = 52.8 ± 10.02 times per day) than age-matched rats housed in the semi-naturalistic condition (*t*_5.3_ = −4.56, *p* = 0.0052; these are corrected Satterthwaite degrees of freedom).

## 4. Discussion

Rats' propensity to burrow remained constant throughout this study at an average frequency of 30 times per day for a total of 20–30 min per day. That rats maintained stable burrowing levels, despite becoming progressively less active, may indicate that rats' demand for burrowing is inelastic [33], suggesting that burrowing is particularly important to rats. Future studies could investigate this idea further, for example by measuring the amount of work rats are willing to perform to gain access to a burrowing substrate.

Burrowing leads to the formation of a burrow, which is crucial for rat survival in the wild. Burrows offer shelter from predators, from light and from the elements, and rats use burrows extensively for sleeping, eating and storing food, and raising their young [15,16]. Burrows can also be advantageous in a laboratory [44]. Retreating into a burrow allows rats to withdraw from perceived threats, such as unfamiliar humans or loud noises [45]; to shelter them from light, which is aversive to rats and leads to retinal atrophy and blindness at levels commonly used in laboratories [46,47]; and to regulate ambient temperature, which in most laboratories is likely to be below rats' thermoneutral zone [48].

This study did not examine whether it is burrowing *per se* that is important to rats, or the functional consequences of burrowing (i.e. having a burrow). Laboratory gerbils, who are prone to developing stereotypic digging, dig much less if an adequate artificial burrow is provided [49]. In our study, cages were furnished with artificial shelters in the form of PVC pipes, but there is evidence that these open-ended pipes are not regarded as satisfactory shelters [50,51]; therefore, burrowing in the presence of these shelters may not be evidence that burrowing *per se* is important to rats. In laboratory mice, burrowing appears to be important regardless of its functional consequences. In one study, mice continued to work to gain access to burrowing substrate despite increasing cost, and burrowed equally whether the burrows they previously built were left intact or destroyed [52].

Some rats' demeanour as they burrowed—running towards the soil, bounding in and out of the soil as they burrowed and bounding away after a bout—suggests that engaging in this activity was reinforcing. Working towards the goal of achieving or maintaining safety (e.g. by building and maintaining a burrow) may itself be rewarding, independently of having safety. Work in human psychology has shown that central to our sense of well-being is how successful and unsuccessful we are in our pursuit of approach and avoidance goals [53]. Approach goals/motivations can be divided into two types: *promotion* motivation, which aims to attain gains (e.g. securing rewards) and *prevention* motivation, which aims to attain non-losses (e.g. securing and maintaining safety [54]). Individuals can be high on one or both of these motivations, and the strength of these motivations is stable across time [55]. Recent work by Franks and colleagues [56–58] has shown that these principles also apply to rats and cotton-top tamarins. For example, rats were given the opportunity to actively maintain darkness and to contain a manageable threat. Those rats who performed these tasks the most frequently (i.e. those who showed strongest prevention motivation) were also those who had lowest indicators of chronic stress [56]. In a broader sense, there are additional benefits to *taking action* and succeeding in achieving and maintaining safety. According to this view, the provision of an adequate burrow may help prevent negative affective states caused by exposure in the open, but the building and maintaining of the burrow—actively working towards safety—may be enjoyable in itself, and may, therefore, provide opportunities for positive affect. Future studies investigating this idea are warranted.

Climbing decreased with age. Three-month old rats climbed, on average, 75 times per day for a cumulative duration of about 15 min, compared with six times per day for a total of about 1 min for 13-month old rats. Maximum climbing bout duration declined from the order of minutes at 3 months of age, to the order of seconds at 13 months of age. Climbing may have declined in part because of rats' tendency to explore less as they age [31,59,60], but we speculate that declining physical ability played a larger role. Climbing requires muscle strength and coordination, both of which deteriorate with age [32]. One study investigated rats' performance while climbing down a wire mesh pole as a function of age. The authors found distinct differences between rats from the various age groups. Younger rats held onto the pole cautiously and climbed down gradually, sometimes turning around to return to the top and repeating their descent. By contrast, older rats often slid down the pole or even fell, never making their way down in a coordinated, systematic manner, and never climbing back up [61].

Young rats climbed frequently and consistently during the dark period. While climbing *per se* may not be a highly motivated behaviour, its performance does add to the limited behavioural repertoire of a captive rat and as such may be beneficial to rat well-being. Because climbing behaviour decreases as rats age, the ability to perform climbing may be more important to young rats than it is to older rats. In addition, Huck & Price [18] have shown that both wild and domestic female rats climb more than males, so the opportunity to climb may be more important to females.

Standing upright was by far the most commonly expressed behaviour of the four measured here, with average frequencies of 180 times per day in 3-month old rats, declining to 75 times per day at 13 months. The total daily duration of standing upright averaged about 10 min per day in young rats, with the large majority of events lasting 1–3 s. It is worth noting that by our definition, standing upright was only scored when a rat's back was straight (or slightly arched due to over extension) and hind limbs extended. Rats frequently stood at a height that was taller than the 18–20 cm allowed by a standard cage, but with their backs minimally curved or hind limbs not fully extended, but these occurrences were not recorded because they did not meet the criteria for upright standing.

As with climbing, the frequency and duration of upright standing decreased as rats aged. Because most occurrences of upright standing were probably exploratory, this behaviour may have decreased as a function of lower exploratory behaviour associated with ageing [31,59,60]. Standing upright does not require particular physical prowess, so declining physical fitness is less likely to have been an important cause for lower expression.

Upright standing is widely expressed even in older rats. It has been suggested that an animals' species-specific forms of kinesis (including stretching and straightening of the back and extending of the limbs) is one of eight systems of behaviour forming the broad basis of animal health and behavioural needs [62]. According to this view, rats' ability to stand upright is an inherent component of their welfare.

Rats housed in the semi-naturalistic cages maintained a consistent daily frequency and duration of lateral stretching as they became older, suggesting that there may be a stable, optimal level of stretching in freely moving rats in this housing system. The fact that semi-naturalistic-housed rats stretched in an upright position when both lateral and upright stretching were possible indicates that there may be advantages to upright stretching. Lateral stretching in standard cages was only scored in 13-month old rats, partly because video scoring was extremely time-consuming, and partly because evidence from semi-naturalistic-housed rats suggested that levels of stretching were relatively stable across time. At this one point in time (13 months of age) standard-housed rats stretched approximately eight times more often than 13-month old rats housed in the semi-naturalistic cages, at a mean frequency of 53 versus 6 times per day (although some lateral stretching in semi-naturalistic-housed rats probably occurred inside their burrows and out of view). This result supports our hypothesis that standard-housed rats performed more lateral stretches to compensate for the inability to stretch upright. However, rats in the semi-naturalistic condition stretched upright about 7–15 times per day (10–20% of the 73 bouts of upright standing in 13-month old rats), so the combined amount of lateral and upright stretching in the semi-naturalistic cages (approx. 13–21 versus 53 in the standard cages) is still far below the amount of lateral stretching observed in the standard cages, suggesting that compensation for inability to stretch upright is not the only motivation driving high rates of lateral stretching. The scientific literature on stretching contends that stretching is a peri-somnolent phenomenon (occurring before or after sleep) but is also expressed in response to stiffness caused by extended periods of immobility, positional stress and sub-optimal movements [42,63]. Rats housed in standard laboratory cages are sedentary [64], having about 0.1 m^2^ floor space for moving around. In the semi-naturalistic cages, rats had 1.7 m^2^ floor space between the different levels; these rats also burrowed and climbed. We suggest that the much higher frequency of lateral stretching in standard-housed rats indicates that standard-housed rats were also using this behaviour to compensate for their generally low levels of mobility. Our measures of lateral stretching were only performed in 13-month old rats, and more age-related data are warranted.

There is evidence that rats reared in the semi-naturalistic environment had better welfare than rats reared in the standard cages [65]. When these rats were 19 months old, they were tested in an anticipatory behaviour test that assessed differences in reward sensitivity between animals reared in the two housing conditions; reward sensitivity is related to an individual's subjective evaluations of his or her internal state [66–68]. Results from this study suggested that standard-housed rats were experiencing poorer welfare compared with the semi-naturalistic-housed rats. There were several differences between the two housing conditions; for example, rats in the semi-naturalistic environment were able to display a larger behavioural repertoire, including burrowing, climbing and upright standing; they were housed in a larger social group; and they had regular access to healthy treats. Which of these factors, or combination of factors, contributed to differences in welfare remains to be tested.

## 5. Conclusion

Laboratory rats reared in an environment that allowed them to burrow, climb and stand upright performed these behaviours consistently throughout the day, and well into adulthood. Burrowing and upright standing appeared to be especially important to rats given the frequency and consistency with which these behaviours were performed. Rats housed in standard laboratory cages were unable to perform these behaviours. Perhaps in compensation for the inability to stand upright, the standard-housed rats engaged in more lateral stretching. This stretching might also be a corrective response to stiffness and positional stress associated with restricted movements in standard cages. These findings suggest that standard laboratory cages interfere with important natural behaviours, and this is likely to compromise rat welfare. On this basis, we suggest that research addressing the direct consequences of preventing, or benefits of allowing, rats to perform these behaviours is warranted.

## Supplementary Material

Electronic supplementary material captions

## Data accessibility

All data are available in a permanent repository on figshare at: https://figshare.com/articles/Datasets_for_article_on_burrowing_climbing_and_upright_standing_in_the_laboratory_rat/3438251; (doi:10.6084/m9.figshare.3438251).
