# Correction to ‘The importance of burrowing, climbing and standing upright for laboratory rats’

**DOI:** 10.1098/rsos.160984

**Published:** 2017-01-04

**Authors:** I. Joanna Makowska, Daniel M. Weary

The caption for figure 2 is incorrect. It should read:

Figure 2. LS mean ± s.e.m. frequency (*a*) and duration (*b*) of burrowing, climbing and upright standing per day per rat at three, eight and 13 months of age in semi-naturalistic cages. Data are mean ± s.e. based on four cages housing five rats at three months, and six cages housing five (*n* = 4) or four (*n* = 2) rats at eight and 13 months; *p *<* 0.05 and ***p *<* 0.001.

