# Supplementary material for: The importance of burrowing, climbing and standing upright for laboratory rats
Source: R Soc Open Sci. 2016 Jun 29;3(6):160136. doi: 10.1098/rsos.160136 (PMC4929907; doi:10.1098/rsos.160136)
Supplement: Electronic supplementary material captions [file rsos160136supp1.docx]

**Electronic supplementary material captions**

**Video S1. Video examples of climbing in the semi-naturalistic environment.**

Climbing can be seen at 2-15 s and 26-29 s. The video was shot with a high definition camcorder (Canon HD10, Japan; 25 frames/s) under low-pressure sodium lighting during the dark phase of the light cycle, when rats were 9 months old.

*Video S1 can be viewed at:* <https://youtu.be/ALg2emQyouE>

**Video S2. Video examples of burrowing and upright standing in the semi-naturalistic environment.**

Burrowing can be seen at 0:02-1:01 min; standing upright can be seen at 23-24 s, 25-26 s, and 32-45 s. The video was shot with a high definition camcorder (Canon HD10, Japan; 25 frames/s) under low-pressure sodium lighting during the dark phase of the light cycle, when rats were 9 months old.

*Video S2 can be viewed at:* <https://youtu.be/bOAAbgVq66E>

**Video S3. Video examples of standing upright and climbing in the semi-naturalistic environment.**

Standing upright can be seen at 3-6 s, 5-6 s, 7-8 s, 8-11 s, and 15-16 s; climbing can be seen at 11-13 s. The video was shot with a high definition camcorder (Canon HD10, Japan; 25 frames/s) under low-pressure sodium lighting during the dark phase of the light cycle, when rats were 9 months old.

*Video S3 can be viewed at:* <https://youtu.be/S-WD7q1QX8w>

**Video S4. Video examples of standing upright and lateral stretching in the semi-naturalistic environment.**

Standing upright can be seen at 2-8 s and 12-14 s; lateral stretching can be seen at 8-9 s. The video was shot with a high definition camcorder (Canon HD10, Japan; 25 frames/s) under low-pressure sodium lighting during the dark phase of the light cycle, when rats were 9 months old.

*Video S4 can be viewed at:* <https://youtu.be/EOjqYxsnN9o>
